# Diabetes, Alzheimer's Disease Risk Factors, and the Cafeteria Diet: A Comprehensive Review

**DOI:** 10.2174/011570159X384737250626094315

**Published:** 2025-07-08

**Authors:** Md Abubakar, Aditi Giri, Falguni Goel, Manshad Khan, Janmejay Gupta, Daksh Kumar, Monika Kaushik, Sachchida Nand Rai, Nitesh Kumar

**Affiliations:** 1 Department of Pharmacology and Toxicology, National Institute of Pharmaceutical Education and Research, Hajipur Export Promotions Industrial Park (EPIP), Industrial Area, Hajipur, Vaishali, 844102, Bihar, India;; 2 Manipal School of Life Sciences, Manipal, Karnataka, India;; 3 Department of Pharmaceutical Technology, Meerut Institute of Engineering and Technology, Meerut, India;; 4 Department of Pharmacology, Amity Institute of Pharmacy, Amity University Madhya Pradesh, Gwalior, Madhya Pradesh, India;; 5 Centre of Experimental Medicine & Surgery, Institute of Medical Sciences, Banaras Hindu University, Varanasi, 221005, India

**Keywords:** Alzheimer's disease, amyloid-beta accumulation, cafeteria diet, metabolic disease, tau hyperphosphorylation, type 2 diabetes mellitus

## Abstract

Alzheimer's disease (AD) is a progressive neurodegenerative disorder with multifaceted risk factors, including diet and metabolic dysfunction. The rising prevalence of AD and diabetes has drawn attention to their shared pathophysiological mechanisms. The “cafeteria diet,” characterized by high-fat, high-sugar, and energy-dense foods, has emerged as a significant contributor to metabolic dysfunctions, including obesity and insulin resistance, which are risk factors for both diabetes and neurodegenerative diseases. This study explores the effects of the cafeteria diet on cognitive impairment, AD pathology, and its potential role in exacerbating diabetes-related neurological complications. Animal models were subjected to cafeteria diets, mimicking human dietary patterns, to investigate changes in brain structure, amyloid-beta accumulation, tau hyperphosphorylation, and cognitive function. Additionally, metabolic profiling demonstrated the development of insulin resistance and other hallmarks of diabetes, which were closely correlated with the severity of cognitive deficits. Neuropathological analyses revealed exacerbated amyloid-beta accumulation and increased neuroinflammation, linking dietary-induced diabetes to AD pathophysiology. These findings underscore the critical role of dietary habits in modulating the risk and progression of AD, highlighting the importance of interventions targeting metabolic health to mitigate cognitive decline. This study emphasizes the need for further research to unravel the molecular mechanisms underlying the diet-diabetes-AD axis and develop targeted therapeutic strategies.

## 1 INTRODUCTION

**Fig. (1):**
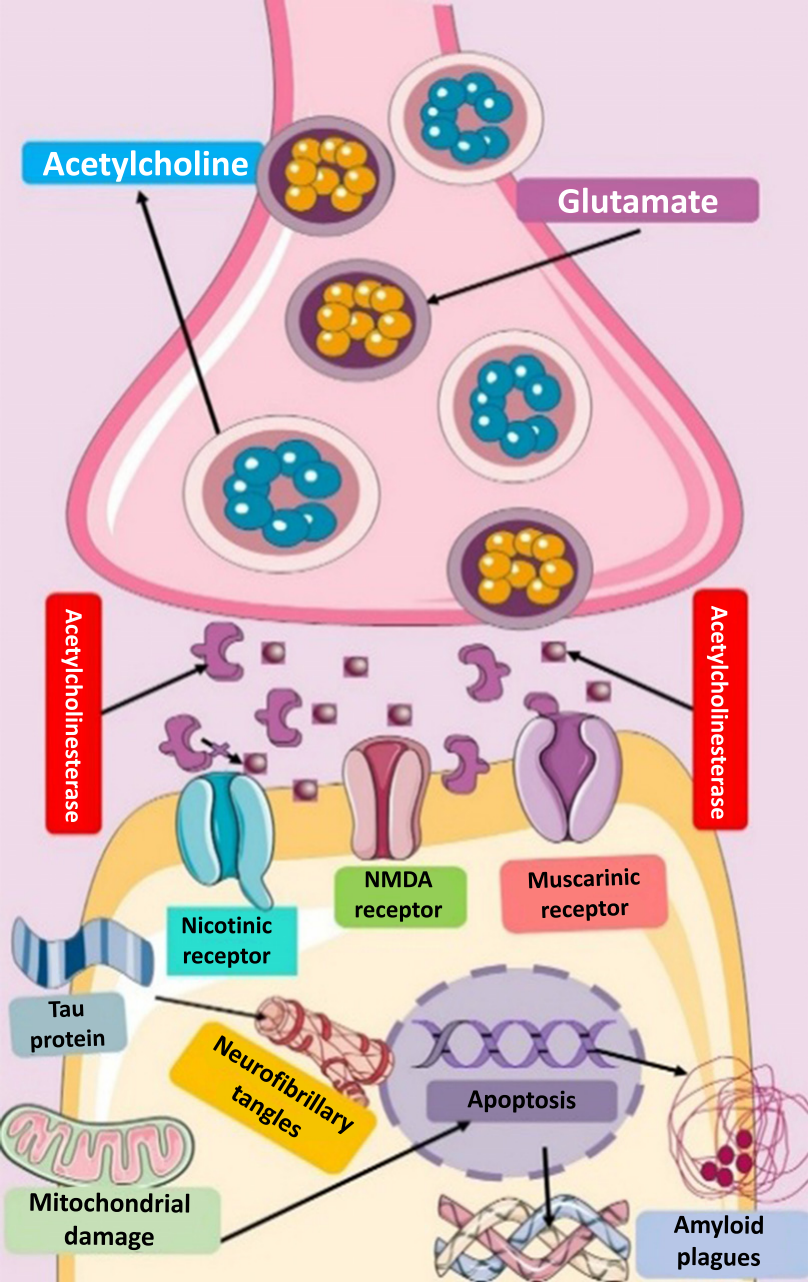
The schematic illustrating AD neurotransmission, showing damaged receptor systems. Due to loss of acetylcholine (ACh) receptors, including nicotinic and muscarinic subtypes, and glutamatergic transmission via NMDA and AMPA receptors, cholinergic signaling is diminished. Also, dysregulated GABAergic and serotonergic pathways cause synaptic dysfunction. The Aβ plaques and tau tangles are key pathogenic components that disrupt receptor function and synaptic connections, contributing to cognitive impairment.

Alzheimer's disease (AD) is the primary cause of progressive dementia and may be the most prevalent neurological illness in the world [1, 2]. AD-associated dementia is due to progressive neuronal insult that leads to injury in the cortical and hippocampal neurons, resulting in a deficiency of cholinergic neurons [3]. With time, mild memory lapses like forgetting recent events or losing things become increasingly worsened [4, 5]. As the disease progresses, some people may find it difficult to solve problems, make decisions, or manage tasks [6]. They often disorient faces, times, and locations. There might be changes in mood or attitude, like anger, apathy, or anxiety. Finding the right words may be difficult, along with following discussions or understanding written and spoken languages [7]. As we see it, the incidence of AD is likely to increase fourfold by 2050. Neurofibrillary tangles (NFTs) consist mainly of hyperphosphorylated tau proteins associating with microtubules [8, 9], and amyloid plaques with Aβ are some of the clinical characteristics of AD (Fig. **1**) [10]. Electron microscope scans of plaques show that they have amyloid fibrils in the brains of AD patients, which show a correlation between the disease and these fibrils [11, 12]. AD clinically manifests as gradual deterioration in memory, language, cognition, judgment, and behaviour. Additional features are dysfunction in mitochondria (MT), imbalances in hormone production [13-15], increased oxidative stress and neuroinflammation, as well as impaired calcium homeostasis. This unique diffraction pattern pertaining only to amyloids is the result of X-ray [16].

Furthermore, the structure of the Aβ fibril can be assessed by solid-state NMR investigations [17]. These studies show that Aβ monomers acquire a β-strand conformation [18]. Somehow, the latest research suggests that if the hydrophilic ends of Aβ monomers make the best possible contact with one another, they combine to form fibrils [19].

The two types of AD are early-onset, where it is called familial AD (FAD), and late-onset, also referred to as sporadic AD [20]. Most early-onset AD patients under the age less than 65 manifest severe symptoms during their early middle age, likely between the ages of 30 and 60 [21]. It is a rare form of AD, accounting for about 5 to 10 percent of all cases. Such patients have inherited it. In early-onset AD, which is often referred to as FAD, mutations of the APP, PSEN1, and PSEN2 genes are involved [22]. Early-onset AD patients may find it difficult to balance work and family, and very quickly develop symptoms; in contrast, late-onset AD usually manifests itself after the age of 65. Although the causes are unknown, behavioural, environmental, or genetic factors might cause them. The APOE ε4 is a prominent genetic risk factor for late-onset AD [20, 23]. The diagnosis is often delayed because it progresses slowly and is often considered a part of aging. Autosomal dominant mutations give rise to early-onset AD, which is a well-known “hereditary disease” [24]. Molecular genetics research has indicated that the course of AD is major influence by Aβ. Mutation or changes at any of the following loci have been shown to cause approximately 5% of cases of AD: at the publicly available amyloid precursor protein (APP) on chromosome 21q21 and PSEN1 on chromosome 14q24, and PSEN2 on chromosome 1q42 [25]. Perhaps most cases of late-onset or sporadic AD demonstrate no clear familial inheritance pattern in etiology. The genetic contribution to AD is very substantial, approximately 70%, similar to schizophrenia's 80%, but higher than that of other complex diseases, including diabetes (40%) as well as PD (30%), according to important research [26]. Any protein can fibrillize into amyloid, forming amyloid fibrils with highly diverse amino acid sequences. However, on closer examination, one might find at the level of secondary structural arrangement that such fibrils differ markedly, having a dominant β-sheet structure. The mutation in PSEN 1/2 or APP gene is responsible for the FAD [27].

Genome-wide association studies help researchers compare millions of genetic variations across a large number, tens of thousands of individuals. Since published by its three GWAS entries for AD in 2009, CLU, CR1, and PICALM have identified three new risk genetic factors for AD [28]. This was regarded as a big advance in AD science. The first event leading to AD is the formation of the Aβ peptide. In addition, excessive phosphorylation of tau impairs its ability to bind to and support microtubules and forms NFTs [28]. These two conditions lead to neuronal degeneration. The amyloidogenic processing occurs when β-secretase cleaves the APP protein rather than α-secretase, and again by the cleavage of γ-secretase. Among these, lifestyle and dietary habits have emerged as critical areas of investigation [29, 30]. The modern “cafeteria diet,” characterized by high intake of processed foods, saturated fats, and refined sugars, mirrors dietary patterns commonly associated with Western lifestyles and has been implicated in the development of various chronic diseases. In parallel, diabetes, particularly T2DM, has been consistently linked to an increased risk of AD, with shared pathological mechanisms such as insulin resistance, chronic inflammation, and oxidative stress [31, 32]. Given that the cafeteria diet often leads to obesity and metabolic dysfunctions that predispose individuals to diabetes, it presents a compelling area of study in the context of AD [33].

Among nutritious meal plans, the MedDiet stands out for its anti-cardiovascular benefits. The MedDiet significantly reduces the likelihood of AD and slows down the progressive memory loss that comes with age, according to a rising number of recent research studies [34, 35]. The MedDiet has the potential to improve brain function by reducing CVS complications such as low cholesterol levels, improper glucose metabolic rate, and high blood pressure; it could additionally minimize the buildup of cerebral Aβ in older individuals and improper glucose metabolic rate. Still, nobody knows for sure what causes these consequences [36-40]. A potential explanation for the beneficial effects of the Ketogenic diet on experimental models' cognitive function might be the formation of β-hydroxybutyrate (β-HB), which could hinder the entry of Aβ_1-42_ into nerve cells, among other mechanisms, shield the hippocampus from Aβ neurological damage, suppress PPAR-γ activation that decreases neuroinflammation, and lessen the development of abnormalities in tau and Aβ buildup by encouraging the elimination of Aβ_1-40_ *via* higher amounts of P-gp, PICALM, and LRP [41-50].

It is becoming more and more apparent that obesity is linked to mental health. Loss of muscle mass and fat during the middle years increases the likelihood of AD and vascular dementia thereafter. Worldwide diagnostic assessments have linked obesity to worse verbal proficiency, perception, and accomplishments. The evidence suggests that cognitive abilities and obesity are not directly connected [51, 52]. Researchers found that individuals with a BMI of 25 or above, spanning the ages of 22-82, performed worse on cognitive ability tests compared to those with an average weight and variances in the results of attentiveness tests. This indicates that, regardless of age, there is a correlation between a higher BMI and worse cognitive function. Several studies comparing obese adolescents to typically developing children found changes in paying attention and cognitive switching [53-55].

Neuroinflammation, insulin resistance, aberrant lipid metabolism, oxidative stress, and altered lipid profiles are all interrelated with cognitive impairment. There is an intricate connection between neurological inflammation and oxidative stress, as these are two key components in the pathophysiology of neurological conditions. ROS and RNS may amplify the pathways of intracellular signaling and therefore promote the production of proinflammatory molecules. In contrast, ROS are generated by active chemicals secreted by inflammatory cells [56, 57]. Transient neural inflammation may aid in protein plaque removal, neuronal equilibrium protection, cell regeneration, and growth promotion; however, persistent inflammation can exacerbate damage to neurons [58]. In AD, changes in the elimination of amyloidosis plaques cause dementia, neural inflammation, and degenerative illness. These symptoms are brought about by aberrant glial cell stimulation. The persistent inflammation caused by obesity affects the brain's CNS, promotes inflammation in the periphery, and decreases BBB susceptibility. In diabetic individuals, enhanced mediators of inflammation activate neutrophils and microglia in brain tissue, triggering neurological inflammation [59, 60]. The SCFAs generated by intestinal microbes from indigestible food fibers are another potential pathway for the gut microbiota to link to immunological diseases and dementia. These seem to regulate the intensity of the intestinal barrier, enhance metabolism within cells, and have substantial immune-regulating action; they are also important intracellular energy sources. In experimental and mouse models, early SCFA administration enhanced interaction between astrocytes and neurons, alleviated cognitive decline by decreasing Aβ deposition, and reduced excessive tau phosphorylation. It also improved neurons' defense against oxidative damage. Although SCFAs have shown neuroprotective attributes in an animal model of AD, the exact mechanisms by which these effects work remain unclear [61-63].

This review delves into the relevance of resistance to insulin, poor glucose utilization in obesity, and mental health, as well as the impact of antioxidant-rich diets in reducing the severity of AD and the effect of cafeteria nutritional supplement compositions upon this interplay. Our main emphasis is on the impact of nutrition and obesity on mental function, as well as the role of oxidative damage in exacerbating obesity and AD pathogenesis. However, this study provides a few instances from both preclinical and clinical settings. There has also been discussion of the role of wealth in antioxidant diets and other therapeutic diets in exacerbating illness conditions. This study reviewed the literature using PubMed, along with additional resources like Scopus and Google Scholar. The usefulness and beneficial results of the article were the deciding factors in its selection. In addition to the cited works, the following topics are covered in the references: AD pathophysiology; obesity and AD interlink pathophysiology; cognitive impairment and the prevalence of obesity; an antioxidant-rich nutrition that decreases mental abnormalities; improved insulin sensitivity; neurological function; neuroprotective effects of AD severity; and neuroscience.

## 2 AD AS A METABOLIC DISEASE AND T2DM

**Fig. (2):**
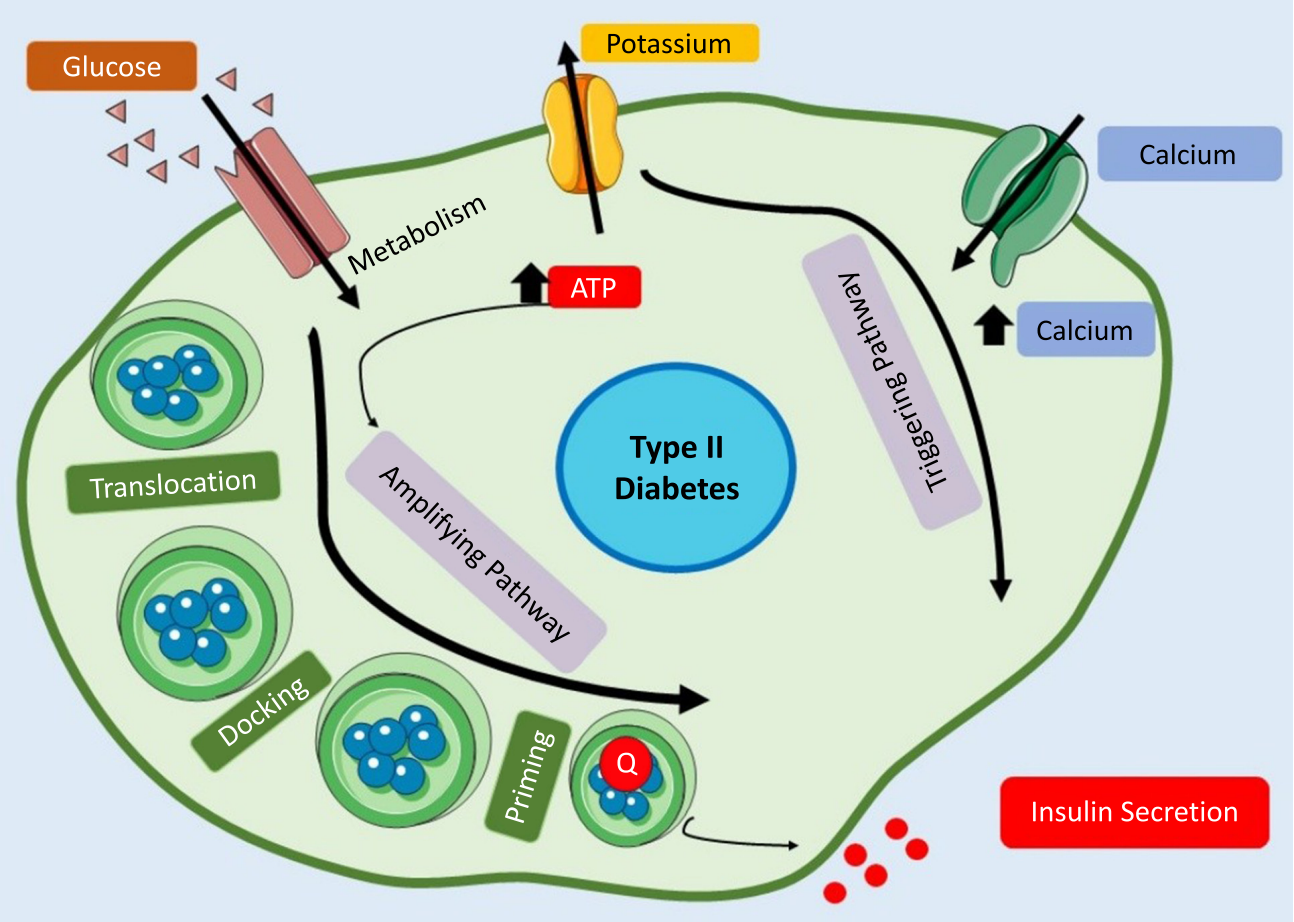
Pathophysiology of T2DM. This figure shows the type 2 diabetes pathophysiology's glucose and potassium regulating molecular processes. Insulin resistance in peripheral tissues impairs the GLUT4 transporter glucose absorption, causing hyperglycemia. Insulin secretion is affected by dysregulated potassium channels, including ATP-sensitive potassium (K) channels in pancreatic beta cells. Chronic hyperglycemia and free fatty acids worsen beta-cell dysfunction, oxidative stress, and inflammation, advancing illness. Impaired insulin receptor signaling, diminished PI3K-Akt activity, and potassium's function in insulin exocytosis, membrane potential are key routes.

AD is a progressive neurodegenerative disorder characterized by cognitive decline, memory impairment, and behavioural changes. Traditionally, AD has been classified as a disease primarily affecting the brain's neurons [64]. However, there is increasing evidence suggesting that AD may also be a metabolic disease, given its significant disruptions in brain metabolism, particularly glucose metabolism (Fig. **2**). The relationship between metabolic dysfunction and neurodegeneration in AD provides new insights into its pathophysiology and opens avenues for potential therapeutic strategies (Table **1**). Table **1** lists various aspects of food and the manner in which they contribute to Alzheimer's disease (such as neuroinflammation, insulin resistance, cognitive impairment, and tau aggregation), as well as some instances of food components that mitigate these symptoms [65, 66]. The brain is highly dependent on glucose as its primary energy source. In AD, one of the earliest and most profound changes is the reduced uptake and utilization of glucose (Fig. **2**) in affected brain regions, such as the hippocampus and cortex [67]. This impairment in glucose metabolism has been found to occur even before the appearance of cognitive symptoms, indicating that changes in brain metabolism might be an early occurrence in the course of AD (Table **1**). PET scans with 18F-FDG (fluorodeoxyglucose) have revealed that glucose metabolism is highly diminished in areas involved by AD, especially during the initial stages of the disease [68]. This condition in AD is associated with impaired glucose metabolism and MT dysfunction (Table **1**).

In most cases, mitochondria produce most of the energy required for the cell in the form of ATP. MT dysfunction compromises the energy production process. This leads to oxidative stress and cellular damage that accelerate neurodegeneration [69, 70]. There is a growing body of evidence indicating that insulin resistance may be an important factor in the development of AD (Table **1**). Insulin, a hormone produced by the pancreas, plays a crucial role in regulating glucose levels in the blood and facilitating glucose uptake into cells (Fig. **2**). In AD, the brain has less sensitivity to insulin, which translates into “brain insulin resistance.” This dysfunction impairs the ability of the brain to utilize glucose effectively. This condition exacerbates further cognitive decline (Table **1**) [71].

**Fig. (3):**
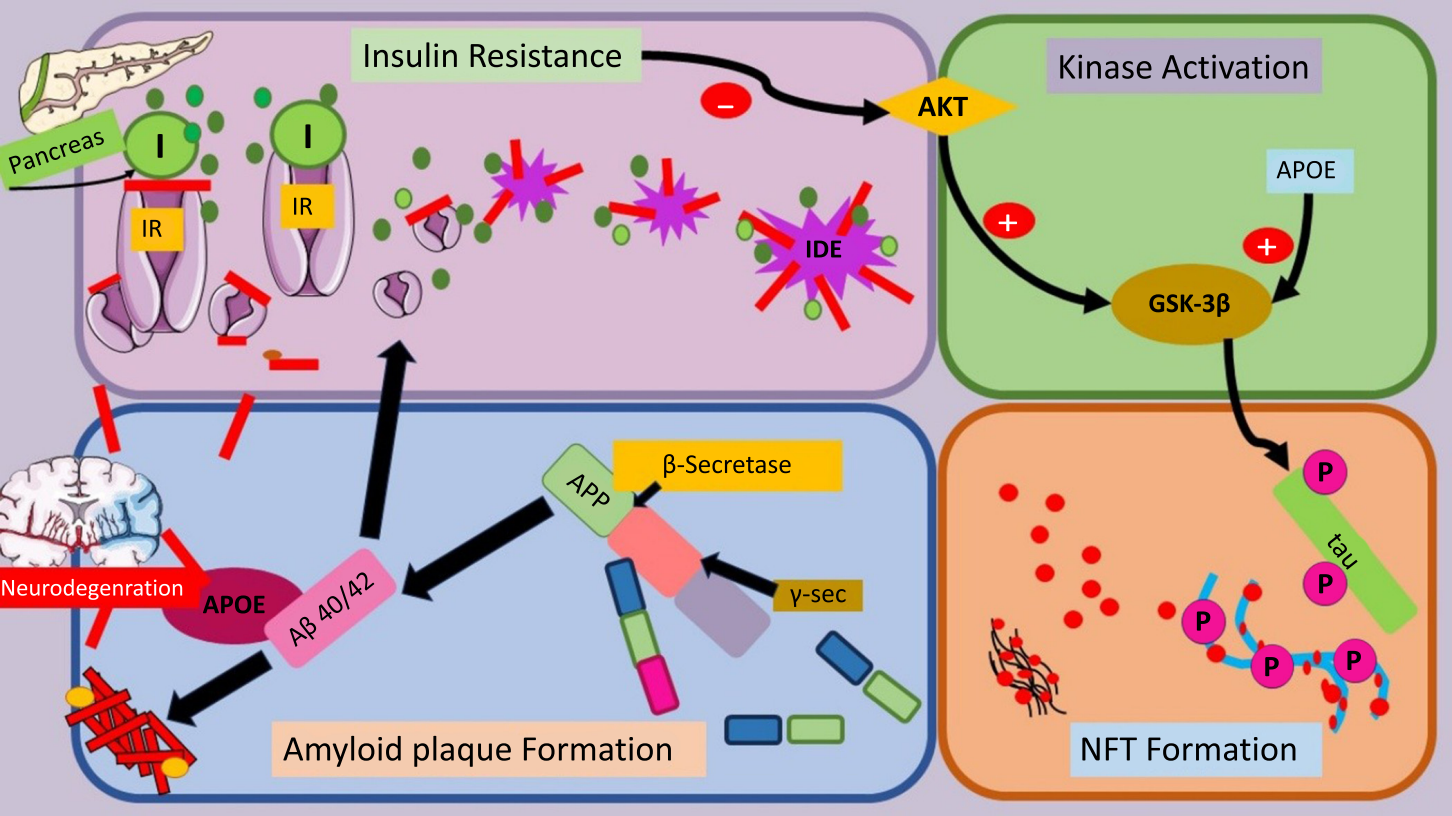
Alzheimer's disease and connection with diabetes mellitus. Emphasizing the role of kinase activation, insulin resistance, and their contributions to neurodegenerative processes in linking AD and diabetes mellitus, the linked pathways emphasize tau hyperphosphorylation and NFTs generation follow from increased kinase hyperactivation brought on by insulin resistance. Concurrent with decreased insulin signaling, Aβ aggregation is encouraged, leading to amyloid plaque deposition. These pathogenic characteristics cooperate to cause cognitive loss, therefore highlighting the intricate interaction between metabolic malfunction and the course of AD.

Insulin resistance has become increasingly recognized as an important risk factor for AD, a common feature of T2DM. The body's cells are less sensitive to insulin in people who have insulin resistance. As a result, blood levels of both insulin and glucose are elevated. The amyloid plaques, a major pathological characteristic of AD, have been linked to this chronic hyperinsulinemia (Fig. **3**). According to research, Aβ peptides build up in the brain as a result of insulin resistance, and these peptides can create plaques that impair neuronal function. The association between T2DM and AD is also alarming. Individuals with diabetes are at increased risk of developing AD, and the degree of cognitive impairment often parallels the level of insulin resistance. The link between diabetes and AD involves complex mechanisms involving metabolic and inflammatory pathways. Hyperglycemia and insulin resistance could facilitate Aβ accumulation, and inflammation secondary to insulin resistance might further augment neuronal injury (Fig. **3**) [71-73]. *In vitro* and *in vivo* studies on animals have contributed enormously to the knowledge about metabolism in AD. In cultured neurons, scientists have shown that high glucose levels or insulin resistance can cause MT dysfunction and increased oxidative stress [74]. These cellular alterations are consistent with those found in AD and provide evidence for the hypothesis that metabolic disturbances are involved in the pathogenesis of the disease. Studies on neuronal cultures treated with Aβ peptides have demonstrated a disruption in glucose metabolism; this further supports the argument that AD-related pathology is associated with energy dysfunction [75].

Moreover, animal models of AD, such as transgenic mice overexpressing APP, have been studied to understand how metabolic disorders affect the course of the disease. These animals show lower glucose uptake in the brain, which is consistent with findings in human AD patients. These animals have demonstrated that faulty glucose metabolism hastens the formation of amyloid plaques and tau tangles, which are the most important markers of neurodegeneration [76]. The study of rodents with induced insulin resistance or diabetes has also proved that these animals have an increased susceptibility to AD-like pathology, such as the deposition of amyloid plaques and cognitive decline [77, 78]. The repositioning of AD as a metabolic disorder has led to further investigation into potential therapeutic avenues focused on enhancing brain metabolism. Interventions to improve insulin signaling have included insulin sensitizers, like metformin, in preclinical models and are under active investigation in clinical trials. Additionally, strategies aimed at improving MT function, reducing oxidative stress, and enhancing glucose metabolism in the brain are being explored as potential treatments for AD. For example, compounds like ketogenic diets or ketone esters have been proposed to improve brain energy metabolism, as the brain can utilize ketones as an alternative energy source when glucose metabolism is impaired [79].

The microbiota of humans is dominated by five phyla: *Firmicutes, Bacteroidetes, Actinobacteria, Proteobacteria*, and *Verrucomicrobia. Firmicutes* and *Bacteroidetes* make up 90% of them. A healthy gut microbiota is shown by a higher ratio of beneficial to harmful bacterial species, which is linked to better host health and general happiness. Conversely, an imbalance promoting harmful bacteria can exacerbate existing illnesses and the development of diseases such as obesity [80]. Microbes produce SCFAs, which are essential to the host's health. Illustrations of these SCFAs are butyrate, propionate, and acetate. These SCFAs mainly aid in preserving the integrity of the gut epithelial barrier by interacting with GPR-41 and GPR-43. Dysbiosis can decrease SCFA synthesis and interfere with barrier permeability. Additionally, gram-negative bacteria found in the stomach create LPS, a substance that can enter the bloodstream and cause chronic inflammation [81, 82].

## 3 DEMENTIA AND DYSPHAGIA

Dysphagia is a frequent sign of AD, especially in the later stages of the illness, but sometimes in its earliest stages, and dysphagia of the oropharynx is a common symptom of AD and related dementias (ADRD). However, dysphagia-related aspiration may lead to pneumonia [83, 84]. There was an additional 2.9-fold increase in the probability of pneumonia mortality in AD patients in comparison to patients with autopsy-confirmed cognitive impairment. Upon dysphagia monitoring, the chance of pneumonia-related death was twice as high in these people. Medical therapy for dysphagia must consider the patient's current drug status, the severity of their dementia, the patient's original pattern, and any potential problems. The dysphagia typically results from issues with either the oesophagus or the oropharynx and is linked to various forms of dementia. Eating difficulties create barriers to eating habits and increase the risk of weight loss, thirst, and malnutrition. Food visual recognition problems, swallowing and feeding difficulties, and oral-tactile agnosia all contribute to oral-phase dysphagia. Another way to look at it is aspiration before, during, and/or after swallowing in the pharyngeal phase of dysphagia. Studies have repeatedly demonstrated that aspiration pneumonia raises mortality rates among dementia patients. A vicious cycle of functional impairments, worsening memory loss, and swallowing difficulties culminates in malnutrition, lack of water, and diminished hunger. Individuals who have trouble swallowing, cognitive impairment, or any of the other linked eating disorders are at an increased likelihood of pneumonia and mortality if they do not eat adequately. For individuals with AD to get tailored therapy, a correct diagnosis of dysphagia is essential [85-88].

## 4 CAFETERIA DIET AND CONNECTION WITH AD

**Fig. (4):**
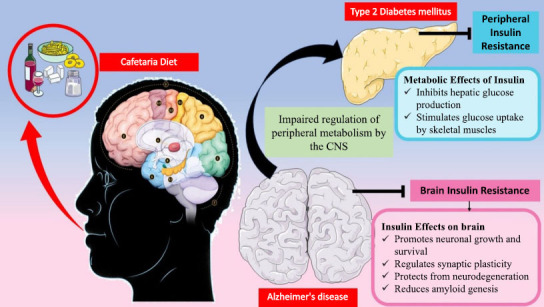
Effects of the cafeteria diet on AD and its association with diabetes. Impact of the cafeteria diet on pathology of AD and related with diabetes. The figure shows how a high-fat, high-sugar cafeteria diet affects cognitive impairment, amyloid-beta deposition, and tau hyperphosphorylation, synaptic plasticity and qualities of AD most importantly. It also emphasizes how the diet aggravates insulin resistance and hyperglycemia, therefore tying metabolic dysfunction with AD progression.

A regular cafeteria meal has been linked to cognitive deficits in AD studies. Oxidative stress in the hippocampus can occur from a cafeteria diet, which can lead to memory loss and a deterioration in cognitive function (Fig. **4**). The hippocampus is an important region affected by Alzheimer's-like conditions. Such a diet is considered a risk factor for metabolic disorders related to cognitive impairment in AD patients. A diet full of nutrients may prevent AD. Observations have shown that a diet from a cafeteria is full of unhealthy and calorie-rich food products that are detrimental to cognitive function (Table **2**), which mentions the impact of the Western and Mediterranean diets on AD [33]. Experiments conducted on animals have shown that diets, which are rich in calories and commonly consumed in a cafeteria, may be devastating in young rats. This diet is hypothesized to be responsible for the metabolic abnormalities associated with an Alzheimer's patient’s cognitive difficulties. It appears that consuming a diet common to a cafeteria, rich in processed carbohydrates and saturated fats, impacts negatively on cognition (Table **2**) [89]. Consequently, consuming lunch at the cafeteria could result in memory loss and cognitive deterioration; this explains why nutrition, if healthy, becomes critical in achieving optimum cognitive functionality [90]. In both industrialized and emerging nations, this suggests that a growing percentage of people eat enough food that is heavy in processed sugar as well as saturated oils [91]. Severe physiological including cardiovascular conditions, increased body weight, and overweight, are all possible outcomes of these eating habits. Several longitudinal as well as cross-sectional investigations have revealed a correlation between high-energy food intake and being overweight, but this also raises the risk of dementia and cognitive decline. Numerous studies have also shown that rats' cognitive capacities are negatively impacted by diets heavy in fat and sugar [92]. In many of the existing paradigms for evaluating cognitive ability, animals are motivated to participate in activities by food. An obese person's appetite may be impacted by changes in the levels of natural hormones, including GLP-1, FGF21, or leptin. The CAF diet increased the effect of oxidative stress on lipids and proteins in the mice's heart, kidneys and hippocampus [93, 94].

## 5 IMPACT OF DIET AND OBESITY ON COGNITIVE FUNCTION

Obesity has been proven to be strongly associated with an elevated risk of AD in large-scale population investigations [100]. There is a continuous negative correlation between BMI, along with cognitive skills such as mental agility and episodic memory, and overweight and metabolic disorders speed up ageing processes in neurons and glial cells, including kinds of brain damage. Memory, intellect, rapid processing, attention, and mental agility are among the many areas of cognition that are markedly influenced by obesity-related dementia [101, 102]. However, obesity-related cognitive impairments can make weight management more challenging, making it harder for people to reach and sustain their weight loss objectives.

According to research, people who are found initially highly impulsive and are poor in cognitive flexibility appear to lose less weight through medically supervised weight-loss programs after 8 weeks. Also, cognitive training program interventions can be incorporated into weight loss to improve inhibition recently; according to the latest meta-analyses, these have been shown to result in considerably improved eating control and weight loss in obese individuals [103]. Whereas it is impossible to separate the individual effects of poor nutrition quality from the cognitive decline brought on by obesity in human studies, some population studies provide evidence. For example, studies across populations have shown that elderly people without dementia who eat high-calorie diets likely have twice as much mild cognitive impairment [104, 105]. How fast and dependent the relations between obesity and cognitive decline on eating habits were rendered complex and observationally deeper from different angles. The evidence continues to accumulate to reinforce the need to counter weight gain along with better nutrition as two avenues to lessen risks for cognitive decline and improve cognition in a wide population base [106].

## 6 OXIDATIVE STRESS, OBESITY, AND AD

The diet plays a very significant role in lifestyle disorders such as overweight, diabetes, hypertension, and CVDs. Diets significantly different from the western diet would include seafood with omega-3 fatty acids and far greater amounts of polyunsaturated fatty acids, used as high cholesterol, saturated fats, and simple sugars [107]. A high diet of polyunsaturated or monounsaturated lipids would be linked to a decreased risk of AD (Fig. **4**) and memory loss, but a high intake of trans fat and saturated fatty acids would increase the risk. Along with such problems, the very obese individuals in middle age are considered to have the highest chances of developing dementia later, with the normal aging-related cognitive decline getting aggravated by the dietary pattern typical of a Western culture [108-111].

There is an evident correlation between oxidative stress and obesity in the onset and progression of AD. Oxidative stress refers to a state leading to destruction within the cell that arises from an imbalance in ROS and antioxidants. This exacerbation is made possible by neuroinflammation, which itself is induced by the increased presence of ROS [112]. Obesity worsens these factors by bringing about inflammation and insulin resistance throughout the body. These pro-inflammatory cytokines probably cross the BBB and thus worsen neuroinflammation in obese subjects, culminating in increased oxidative damage. In this context, insulin resistance would impair brain glucose metabolism and interfere with the normal processes to clear Aβ from the brain and has detrimental effects on brain glucose metabolism (Fig. **4**). But strong oxidative stress and the obesity cycle get entangled into a downward cycle that further damages neurons, creating a neurodegenerative pattern typical of AD [113]. Therefore, for such individuals, cognitive deterioration can be hastened because they come, one way or another, to the effects of AD much earlier or more readily as a result of the amplifying interaction of these factors. Animal findings have shown that high-fat diets greatly affect hippocampal-dependent memory and learning, showing a potential connection to the onset of dementia. However, the specific processes involved remain unclear [114, 115]. Thus, habitual eating patterns are now associated with weight gain, obesity and cognitive decline with age, which highlights the very natural stakeholders for nutrition in brain function and health outcomes. The essential part of the damage is at the hippocampus, where the inhibition, formation of episodic memory, and spatial processing take place, which are particularly relevant functions on cognitive decline associated with obese subjects. Impairments of this brain area are expected to be the basis for dysfunctional adaptive eating behaviours and thus the decision-making process related to food choices, which can contribute both to a further increase in weight and a reduced ability to lose it [116]. Understanding these mechanisms of cognitive dysfunction produced by obesity will be exceptionally important to augment the efficacy of weight management initiatives, improve living standards, and relieve stress on global healthcare systems [117].

## 7 COGNITIVE DYSFUNCTION ASSOCIATED WITH OBESITY

Cognitive impairment associated with obesity among human populations is steadily increasing, and evidence is beginning to show that extra weight adversely influences functions in the brain. Specifically, obesity, often with metabolic syndrome and insulin resistance, has been established to be detrimental to many capacities like memory, learning, and executive functioning [118]. For one, excess adipose tissue in an obese person secretes inflammatory cytokines that can diffuse into the brain to initiate chronic neuroinflammation, impairs function by damaging neurons and disrupting their ability to transmit signals. This condition also leads to oxidative stress, furthering neuronal damage and aggravating cognitive decline. Furthermore, insulin resistance, typically associated with obesity, hampers the brain's ability to metabolize glucose effectively, starving neurons of energy and impairing their functioning [119]. It has also been noted that obese individuals have structural changes in the brain, especially a reduction in gray-matter volume, which has been correlated with poorer cognition. These gradually aggravate into more serious cognitive impairments, which could increase the risk of developing AD [120-122].

Obesity has been highlighted in recent studies as a detrimental influence in several cognitive domains, including attention, intelligence, memory, cognitive flexibility, speed of processing in cognitive tasks, and executive function. It is well-established that there is a negative relation between body mass index and both executive function and episodic memory [123-125]. However, the exact nature of the relationship between secret metabolic dysfunction and obesity is still unclear. The negative effects that obesity may have on adults have yet to be fully understood, but are often implied and associated with cognitive dysfunction at midlife and calls of alarm against dementia and age-related cognitive decline [126].

Research in 2022 showed that obesity at midlife is at higher risk of cognitive decline in later years, particularly affecting memory, attention, and executive function. According to the study, those with higher measurements of body mass index (BMI) performed deplorably on cognitive tasks that test memory and decision making. Furthermore, changes in brain structure as a result of obesity [127], such as neuronal atrophy in the hippocampus and frontal regions, have been associated with diminished cognitive abilities. Similarly, a 2023 investigation examined a cohort of South African women, revealing that the ways in which obesity hindered cognitive performance in memory tests, attention tests, and executive function tests were considerably less than that observed in normal-weight individuals. Evidence from long-term studies, however, states that obesity in middle age may increase the risk for neurodegenerative diseases, such as AD, as one ages. These findings highlight the intricate association between obesity and cognitive health, which involves neuroinflammation, oxidative stress, and metabolic changes [128].

Brain health is severely affected by obesity and has effects on global brain volume and white matter connections within the brain. Some current studies show that this thinner cortex tends to exist for people aged under 65 years with an elevated BMI and waist circumference; the effect is seen in particular brain regions such as the hippocampus because thinner cortices have been linked to diminished integrity of white matter and a reduction in the volume of grey matter [129], where it has been identified. Such changes are likely structural bases for performance deficits on cognitive tasks that rely on hippocampal and temporal lobe functionalities. Besides, findings indicate that inflammation is one of the links between obesity and cognitive decline. For instance, it is well known that obesity increases the most types of immune cells (macrophages, T-cells, and microglia). Obesity, therefore, increases the production of many pro-inflammatory cytokines (such as TNF-α, IL-6, IL-1β, and MCP-1), free fatty acids, and chronic low-grade continued inflammation in these peripheral organs that might extend to neuroinflammation. Most likely, the above signals travel through systemic immunological and nutritional signals (Leptin signaling pathway, insulin signaling pathway, ghrelin signaling pathway, GLP-1 pathway, CCK pathway, NPY and AgRP pathway, and melanocortin pathway), can have an impact on food behaviours regulated by the perirhinal cortex, hippocampus, and hypothalamus [130-132]. This series of incidents triggers neuronal death, impairs neurogenesis, and causes synaptic plasticity defects. Eventually, this results in cognitive deterioration and neurodegeneration.

The rising modern eating habits with an increased intake of fats and sugars encourage the hypercaloric conditions that cause many metabolic dysfunctions, such as peripheral insulin resistance. Such diets seem to bring changes in the brain, such that they can cause insulin resistance, MT dysfunction, and increased oxidative stress, increasing an individual's susceptibility to dementia, including AD [133-135]. Alzheimer's disease is related to cognitive decline due to neurotoxicity stemming from plaques of Aβ that lead to oxidative stress and neuronal apoptosis.

There is evidence that oxidative stress is characteristic of ageing and age-related diseases like AD due to an imbalance in the reactivity of ROS production and cellular antioxidant defenses. It has been proposed that reducing oxidative stress with antioxidant treatments, physical activity, and a diet rich in antioxidants can safeguard the brain's MT and delay the onset of AD [136]. These issues are made worse by obesity-associated oxidative stress, which connects obesity to systemic impairments, including kidney impairment, liver disease, along with cardiac problems in addition to cognitive dysfunction. Preventing or reducing cognitive impairment or enhancing overall health might be achieved by interventions that target the harmful oxidative stress linked to obesity [137-139].

## 8 ANTIOXIDANTS

The antioxidants have an essential role in the progression of AD by mitigating oxidative stress, which is thought to contribute to the development of the illness. Reports suggest that antioxidants possess the capacity to hinder or maybe manage AD. Research has demonstrated that extended use of antioxidants can significantly decrease oxidative stress in models of AD, therefore emphasizing their ability to prevent the condition [140]. This study has covered the anticipated results, the small number of naturally occurring antioxidants with neuroprotective activity, and their ability to suppress protein aggregation and lessen the degree of oxidative damage in AD in Table **3** [141-149].

### 8.1 Role of an Antioxidant-rich Diet

Intake of diets that are rich in fats and sweets normally acts as a factor that enhances oxidative stress. Powerful antioxidants present in plant-based supplements could have applications for the prevention and treatment of chronic disorders like obesity [150]. In order to improve our ability to identify and treat AD, it is vital to establish a connection between the genetic, metabolic, physiological, neuro-imaging, and clinical issues associated with AD and the development and advancement of neuropathological lesions [151]. Hence, enhancing our understanding of the biology of these deviations could aid in the diagnosis and treatment of AD.

### 8.2 Gut Microbiota on Cognition and its Importance

AD and related dementias belong to the most widespread and costly medical conditions worldwide. Over the past two decades, there has been a growing realization of the significant influence of the gut microbiota on human health, particularly in the context of neurological disorders. There is substantial evidence indicating that the gut microbiota may have an important impact on the development of dementia, including AD. Moreover, it has been observed that imbalances in the microbial composition of the GIT are associated with a decline in cognitive function [152]. Targeting the gut microbiota by dietary and activity modifications might be a great method to lower the risk of developing dementia. All of the bacteria, viruses, protozoa, and fungi that are found in the GIT, as well as all of their genes, are included in the gut microbiome. *Firmicutes, Bacteroidetes, Actinobacteria,* and *Proteobacteria* are the four main phyla that make up the microbiota that lives in the GIT. They are necessary for the absorption of nutrients and minerals as well as for the synthesis of enzymes, vitamins, amino acids, and neurotransmitters. Metabolites, including SCFAs, butyrate, propionate, acetate, and others, are also produced by a healthy microbial ecosystem. These metabolites support immune system regulation, pathogen defense, and the integrity of the epithelial barrier. A healthy gut microbiome is characterized by a very high level of bacterial diversity, due to symbiosis among pathogenic and non-pathogenic bacteria, as well as host-derived parts like immunoglobulins (IgA, IgM), mucins, and antimicrobial peptides.

An imbalance in the gut microbiota, which is called dysbiosis, is associated with issues related to the immune system, nervous system, and metabolism. Among the materials for possible causes are the medications' side effects, old age, poor diet, infections, as well as increased permeability and inflammation of the intestines. In the enteral environment of dysbiosis, all of these may be adversely affected. These include a breakdown in the gut-brain axis, which is an interconnected network of communication *via* the vagus nerve with the HPA axis, cytokines, and other neurological pathways. A dysbiotic intestinal environment can negatively impact the gut-brain axis, a network of communication that includes the vagus nerve, the HPA axis, cytokines, and other neurological pathways. Among the other possible causes are medication side effects, old age, poor nutrition, and exposure to infections. Increased gut permeability and gut inflammation may also contribute to these. Increased permeability in the intestine, caused by microbial dysbiosis, will release endotoxins, like LPS, into the bloodstream, thus causing an immunological reaction in the CNS. The stomach raises alerts through vagus nerve activation to the brain concerning its dysbiosis condition through the release of proinflammatory cytokines and cortisol, as well as activation of the HPA axis. Prolonged elevation of blood proinflammatory cytokines along with cortisol can damage the BBB shrinkage in the brain, aggravating neuroinflammation; all these may influence mental health and brain functioning.

Microbiota dysbiosis has been associated with cognitive decline in AD patients. A functional and diversified microbial ecosystem is essential for better learning, memory, and behavioural adaptations. Since there is no known cure for dementia, efforts are currently being made to promote good lifestyle choices and change risk factors toward cognitive impairment. Ongoing research will investigate the combined benefits of multiple lifestyle interventions, such as their synergic effect in dietary modifications and physical activity. Long-term dietary practices determine the composition and function of the gut microbiome; however, they can easily be modified with dietary adjustments and physical exercise. Indeed, there are likely relationships between microbiome health, diet, exercise, and cognition. However, scant intervention studies have directly investigated these phenomena, and none have yet focused their entire remit on cognition.

### 8.3 Diet, Cognition, and the Microbiome

The western diet-associated deficiency in cognitive function and a tendency towards dementia is in close association with the fiber-poor, fat-dense, and simple-carbohydrate-rich diet. By contrast, the MedDiet appears to contribute to higher cognition and decreased dementia risks. The MedDiet is generally recognized as a brain-friendly diet: it promotes neurogenesis, lowers oxidative stress and inflammation, and improves neuronal connections. It has consistently been associated with a reduced often prevented risk of developing AD as well as neurodegenerative dementia. One of the mentioned mechanisms by which the diet is thought to work is that it provides a high content of antioxidants, omega-3 fatty acids, and polyphenols that potentially exert anti-inflammatory and neuroprotective properties. Research has shown that adherence to the MedDiet significantly slows cognitive decline, as well as reduces the incidence of dementia. Research has shown that the MedDiet has an ameliorative effect on oxidative stress and inflammation, which are major contributors to AD.

The “Western diet,” on the other hand, emphasizes a diet heavy in processed meals, processed sugars, and fatty foods, which seldom qualify as vegetable or fruit consumption, and has physiological consequences that are at odds with those of obese and insulin-sensitive individuals. Such a lifestyle predisposal effect manifests within metabolic syndrome, increasing the risk of AD. Such a Western diet leads to gut microbiome dysbiosis, which promotes neuroinflammation and catalyzes cognitive decline (Table **4**) [153-159]. The MedDiet has effects on brain health. It proves to be anti-inflammatory, antioxidant, and nutritious in the context of the relative health risks associated with AD risk factors, which are increased by the western diet with its high proportions of processed foods and unhealthy fats. It also highlights the importance of dietary interventions in preventing or delaying the onset of AD (Table **4**).

## CONCLUSION AND FUTURE PERSPECTIVE

The cafeteria diet can be characterized by high intake of fat, sugars, and processed foods, and it has been shown to contribute to the development and progression of AD, as evidenced by its mechanisms, including insulin resistance, oxidative stress, and neuroinflammation. The harmful effect is somewhat analogous to that seen in pathological features of AD, such as Aβ aggregation, tau hyperphosphorylation, and impaired cognitive function [160, 161]. The cafeteria diet's strong ties to obesity and diabetes worsen the effects of both conditions, which create chronic inflammation, MT dysfunction, and further insulin resistance, and thus damage brain welfare further. Synergistic processes, including resistance to insulin and persistent inflammation, underlie the development of metabolic disorders and AD-like pathology. By blocking APP breakdown, a high-glucose environment enhances Aβ formation, and increased amyloid beta buildup and tau phosphorylation are symptoms of insulin resistance, which is caused by aberrant regulation of insulin in the brain. A critical need for preventative dietary and metabolic health measures against AD is highlighted by the interaction underlying neurodegeneration occurrences and metabolic imbalances caused by the diet [72, 162-166]. Beneficial effects in AD patients have been associated with the ketogenic diet, characterized by low carbohydrate intake, moderate protein consumption, and high fat intake. Through lowering neurological inflammation, balancing gut bacteria, increasing mitochondrial activity, and reducing cellular oxidative stress, research suggests that ketogenic diets may enhance cognitive performance in AD [167-170]. The wide variety of microbes seen in dysbiosis, an imbalance in the gut flora, has been associated with a host of health problems, including AD, diabetes, and obesity [171]. When it comes to avoiding chronic illnesses, it appears that multicomponent supplements or dietary habits are more effective than those that focus on just a single or two components. This highlights the necessity for massive, ongoing research investigations that consider various food groups and their compositions [106, 172-175].

In the future, a more thorough study should be conducted linking metabolic derangements as obesity and diabetes, with neurodegenerative diseases. This evidence only demands dietary consideration and lifestyle modification to reduce the risk of future occurrences of AD, potentially [176, 177], especially in individuals at high risk, such as those with a metabolic disease. We can grasp the multifaceted character of the illness by examining its pathophysiological cascades and biological processes. As a result, this study has developed multiple promising medications in both experimental and clinical settings, highlighting the need for further investigation in this area [178]. Potentially helpful in warding off AD is a healthy, well-balanced diet high in nutrition that reduces inflammation and free radical damage [179]. Those who have diabetes must take special care to prevent both extremes of eating inadequately and consuming too much to keep their blood sugar levels under control. An improvement in glucose and blood sugar levels, as well as a possible slowing of degeneration, may result from regular calorie intake assessment [180, 181]. It has been shown that antioxidants from the diet, including nutritional supplements and medicinal products, may effectively treat AD, in addition to regulating environmental triggers that cause oxidative damage. Prevention of age-associated chronic diseases and an increase in longevity duration among older adults may be achieved through nutritional strategies in conjunction with the avoidance of associated risks [182, 183]. Although being overweight increases the likelihood of diabetic cognitive impairment, it is important to monitor the mental health of individuals regularly. Careful oversight, including the use of antioxidants when needed, is required for the treatment of diabetic cognitive problems in those who are overweight or have metabolic impairments [184-188].

Antioxidants found naturally in the body may prevent the generation of free radicals and even influence the development of chemicals that enhance neurons' antioxidant activity. Evidence from extensive research suggests that protective enzymes, such as CAT and SOD, may delay the onset of AD. Furthermore, preclinical investigations have suggested that certain dietary antioxidants may have therapeutic potential. Although *in vivo* trials are crucial for early research, this result may not fully represent preclinical evaluations, and questions remain about the effectiveness of antioxidants in real clinical settings. Preclinical therapy with antioxidant compounds may offer protection against AD, but their use after the fact is ineffective. Unfortunately, there are currently no case-control investigations available, so we don't know how effective the MedDiet is in preventing or slowing the course of AD. Improving neurodegenerative symptoms with dietary modification might be ineffective if initiated later in life, before proper identification. Determining whether dietary prioritization in adolescence or later years has restorative benefits and identifying which aspects of lifelong diet have the greatest influence on cognition in old age requires ongoing case-control research [95, 189, 190].

## STUDY LIMITATIONS

Individuals with T2DM are under-recognized when it comes to mental health testing, and treatment for cognitive decline is very infrequent. Likewise, T2DM and elevated insulin dosages are not routinely assessed in individuals with AD. Rather than addressing the underlying cause of this condition, current therapies aim to alleviate the symptoms and discomfort that come with it. For more information on the genetic and cell-based mechanisms that make these foods beneficial for your health, further study is needed. Our understanding of disease pathways could improve, and new treatment options may emerge, if mitochondrial dysfunction is shown to be a common underlying factor in both diabetes and Alzheimer's disease (AD)-related neurodegeneration. Consequently, present treatment choices for these two progressively and often associated illnesses should be enhanced by treatments established to combat mental retardation caused by diabetes and dementia mediated by AD, which includes mitochondrial malfunction and redox status disequilibrium. A novel therapy possibility for minimizing, curing, and postponing age-related and metabolic-related disorders has emerged with mitochondrial healthcare, which recovers mitochondrial activity and bioenergetic networks in the neurological system. It also offers additional therapies that can be used in conjunction with current treatments.

## Figures and Tables

**Table 1:** Key dietary components and their impact on AD and diabetes.

**Dietary Component**	**Impact on AD Pathology**	**Link to Diabetes**	**Mechanism**
High saturated fat	Increases Aβ plaque formation, tau hyperphosphorylation, and neuroinflammation	Contributes to insulin resistance, leading to impaired glucose metabolism	Disrupts MT function, enhances oxidative stress, and promotes neuroinflammation
Refined sugars (*e.g*., glucose, fructose)	Elevates Aβ plaques and NFTs, impairs memory and learning	Leads to increased blood glucose levels, contributing to insulin resistance and T2DM	High sugar intake induces glycaemic spikes, increases oxidative stress, and accelerates brain aging, promoting cognitive decline
Processed foods (*e.g*., fast food, packaged snacks)	Contributes to obesity and chronic inflammation, exacerbating AD progression	Increases the risk of T2DM by promoting obesity and insulin resistance	High in unhealthy fats, salt, and sugar, processed foods worsen metabolic dysregulation and trigger inflammatory responses in both the brain and body
Trans fats (*e.g*., partially hydrogenated oils)	Promotes neurodegeneration, increases plaque formation, and cognitive decline	Impairs insulin receptor function, worsening insulin resistance and diabetes	Alter cell membrane composition, disrupt cell signaling, and promote inflammation, contributing to both AD and diabetes
High-salt diet (*e.g*., salty snacks, processed meats)	Aggravates cognitive decline by impairing brain blood flow and inducing neurovascular dysfunction	Increases the risk of hypertension, which worsens insulin resistance and diabetes	Disrupts vascular function, impairing cerebral circulation and increasing systemic inflammation
Low-fiber diet (*e.g*., refined grains)	Impacts gut-brain signaling, increasing inflammation and cognitive decline	Contributes to insulin resistance and poor glycaemic control	A lack of dietary fiber promotes gut dysbiosis, increases systemic inflammation, and impairs glucose regulation in both the brain and peripheral tissues
High dairy fat (*e.g*., whole milk, cheese)	Promotes neuroinflammation and tau pathology in the brain	High dairy fat is linked to obesity and insulin resistance, increasing diabetes risk	Trigger inflammation and oxidative stress in both the brain and peripheral tissues
Monounsaturated fat (*e.g*., olive oil, avocados)	May reduce Aβ and improve cognitive function	Supports insulin sensitivity, reducing the risk of T2DM	Promotes healthy lipid profiles, reduces inflammation, and enhances neuronal function
Omega-3 fatty Acids (*e.g*., fish oil, flaxseed)	Reduces Aβ levels, improves memory, and decreases neuroinflammation	Improves insulin sensitivity and reduces inflammation associated with diabetes	Promotes anti-inflammatory pathways, reduces oxidative damage, and improves neuronal function
Polyphenols (*e.g*., flavonoids, curcumin)	Protects against oxidative stress, reduces Aβ accumulation, and inhibits tau aggregation	Helps regulate blood sugar levels and improves insulin sensitivity	As antioxidants, they reduce neuroinflammation and improve glucose metabolism through various signaling pathways

**Table 2:** The role of the Mediterranean diet (MedDiet) and Western diet in AD.

**Diet Type**	**Impact on AD**	**Mechanisms**	**Supporting Studies**	**References**
Mediterranean diet (MedDiet)	Protective	Rich in antioxidants, polyphenols, healthy fats (omega-3), and low in processed foods; supports brain health and reduces inflammation	Studies show a lower risk of cognitive decline and slower AD progression	[95]
	Reduces the risk of cognitive decline in AD	Anti-inflammatory properties, neuroprotective effects from polyphenols (*e.g*., in olive oil and red wine), support gut microbiome	Long-term adherence to the MedDiet is linked to reduced incidence of AD	[96]
Western diet	Exacerbates AD risk	High in saturated fats, sugars, and processed foods; increases oxidative stress, inflammation, and insulin resistance	Increased AD risk is associated with diets high in fat and sugar	[97]
	Promotes cognitive decline	Disrupts the gut-brain axis, increases Aβ aggregation and tau phosphorylation	Dysbiosis in the microbiome is linked to cognitive impairment and AD	[98]
	Associated with metabolic dysfunction	Leads to obesity, insulin resistance, and metabolic syndrome, which are risk factors for AD	Studies show that obesity and metabolic syndrome increase the likelihood of AD development.	[99]

**Table 3:** List of antioxidants, their sources, and DM-induced AD model *in vivo* and *in vitro* results.

**Antioxidant**	**Source**	**Study Type**	**Findings**	**References**
Quercetin	Apples, onions, and berries	*In vitro* and *in vivo*	Demonstrates neuroprotective effects by inhibiting acetylcholinesterase and reducing oxidative stress in AD models	[141]
Myricetin	Berries, grapes, and walnuts	*In vitro* and *in vivo*	Exhibits antioxidant properties, improved glucose uptake, and provides neuroprotection against oxidative stress in neuronal cells	[142]
Epicatechin-3-gallate	Green tea	*In vitro*	Showed inhibition of β-secretase activity, reducing amyloid-beta aggregation associated with AD	[143]
Naringenin	Citrus fruits	*In vivo*	Improves cognitive function and reduces oxidative stress markers in AD-induced animal models	[144]
Apigenin	Parsley, chamomile	*In vitro*	Inhibits tau protein aggregation and exhibits anti-inflammatory effects relevant to AD pathology	[145]
Genistein	Soy products	*In vivo*	Enhance insulin sensitivity and provide neuroprotective effects in diabetic animal models, potentially mitigating AD progression	[146]
Cyanidin 3-O-glucoside	Red berries	*In vitro* and *in vivo*	Reduces oxidative stress and improves cognitive function in AD models; also exhibits anti-diabetic properties by modulating glucose metabolism	[133]
Tropoflavin (7,8-Dihydroxyflavone)	Synthetic flavonoid	*In vivo*	Act as a TrkB receptor agonist, promoting neuroprotection and cognitive improvement in AD models	[147]
Sorghum polyphenols	Sorghum grains	*In vitro* and *in vivo*	Demonstrates neuroprotection through antioxidant, cholinergic, and anti-amyloidogenic pathways in AD models	[148]
*Lactuca sativa* extract	Hydroponic lettuce	*In vitro* and *in vivo*	Exhibits antioxidative, anti-Alzheimer's, and anti-diabetic activities, suggesting therapeutic potential for AD and DM	[149]

**Table 4:** Illustrates the molecular importance of varied diets in mitigating AD severity in several preclinical and clinical studies.

**Study Type**	**Diet Composition**	**Duration of Diet (Age at Start)**	**Species/** **Participants**	**Mechanism/Pathway**	**References**
Preclinical	High-fat, high-sugar diet with processed foods (*e.g*., biscuits, sausage, cheese, sugary drinks)	12-16 weeks (start at 4-6 weeks)	Rodents (rats, mice)	Insulin resistance, increased Aβ deposition, reduced synaptic plasticity, and chronic inflammation in the hippocampus	[153]
Preclinical	Cafeteria diet with refined carbohydrates and saturated fats (*e.g*., chips, cookies, and chocolate)	20 weeks (start at 2 months)	Mice (APP/PS1 transgenic)	Enhances tau hyperphosphorylation, oxidative stress, and MT dysfunction linked to diabetic neuropathy and AD-like cognitive decline	[154]
Preclinical	High-calorie cafeteria diet with sugary beverages and processed snacks	8 weeks (start at 3 months)	Rats	Alteres gut microbiota, disrupted gut-brain axis, increased neuroinflammation, and impaired insulin signaling in the brain	[155]
Preclinical	High-sugar, high-fat diet supplemented with sweetened condensed milk, chips, and processed meats	24 weeks (start at 8 weeks)	Rats	Increased blood glucose, advanced glycation AGEs formation, impaired insulin signaling, and exacerbated APP	[156]
Preclinical	High-fat cafeteria diet with pastries, bacon, and sweetened foods	16 weeks (start at 4 weeks)	Rodents (mice, rats)	Reduces neurotrophic factors (*e.g*., BDNF), heightened inflammatory cytokines (*e.g*., TNF-α, IL-1β), and disrupted blood-brain barrier integrity	[157]
Clinical Studies	Western-style diet with processed foods, sugary snacks, and high saturated fats	Long-term observational studies	Adults (aged 45-70)	Correlation between diet-induced obesity, insulin resistance, and accelerated cognitive decline, with higher alzheimer’s risk in patients with T2DM.	[72]
Clinical Studies	High-fat, high-sugar diet is typical of cafeteria-style eating	Cross-sectional studies	Adults and the elderly	Elevates HbA1c, fasting glucose levels, and impaired glucose tolerance linked to worsened cognitive scores and biomarkers of Alzheimer's (*e.g*., tau, Aβ42 levels)	[158]
Clinical Studies	High-calorie Western diet	Retrospective cohort studies	Elderly (aged > 60)	Association between diet-induced metabolic syndrome, increased amyloid burden on imaging studies, and greater AD progression in diabetic individuals	[159]

## LIST OF ABBREVIATIONS

AD: Alzheimer's Disease
APP: Amyloid Precursor Protein
BMI: Body Mass Index
MT: Mitochondria
NFTs: Neurofibrillary Tangles
β-HB: β-hydroxybutyrate
